# Development of a participatory action research–based intervention to enhance infection prevention and antimicrobial stewardship among European nursing home staff

**DOI:** 10.1093/jacamr/dlag158

**Published:** 2026-08-12

**Authors:** Marie Theut, Jesper Lykkegaard, Valeria Antsupova, Malene Plejdrup Hansen, Tina Marloth, Carl Llor, Ana Garcia-Sangenis, Ramon Monfà, Ana Moragas, Marilena Anastasaki, Greta Tsoulchai, Christos Lionis, Nina Sodja, Davorina Petek, Ria Benko, Andras Balint, Anna Kowalczyk, Lina Jaruseviciene, Helena Glasova, Jozef Glasa, Jette Nygaard Jensen

**Affiliations:** Research Unit for Antibiotic Stewardship and Implementation, Department of Clinical Microbiology, Copenhagen University Hospital—Herlev and Gentofte, Herlev, Denmark; Research Unit for General Practice, Department of Public Health, University of Southern Denmark, Odense, Denmark; Research Unit for General Practice, Department of Public Health, University of Southern Denmark, Odense, Denmark; Department of Clinical Microbiology, Copenhagen University Hospital—Herlev and Gentofte, Herlev, Denmark; Research Unit for General Practice, Department of Public Health, University of Southern Denmark, Odense, Denmark; Center for General Practice, Aalborg University, Gistrup, Denmark; Department of Clinical Microbiology, Copenhagen University Hospital—Herlev and Gentofte, Herlev, Denmark; Institut Català de la Salut, Barcelona, Spain; CIBER de Enfermedades Infecciosas, Instituto de Salud Carlos III, Madrid, Spain; CIBER de Enfermedades Infecciosas, Instituto de Salud Carlos III, Madrid, Spain; Institute for Primary Health Care Research Jordi Gol i Gurina (IDIAPJGol), Barcelona, Spain; Institute for Primary Health Care Research Jordi Gol i Gurina (IDIAPJGol), Barcelona, Spain; Institute for Primary Health Care Research Jordi Gol i Gurina (IDIAPJGol), Barcelona, Spain; Jaume I Health Centre, Catalonian Health Institute, Tarragona, Spain; University Rovira i Virgili, Reus, Spain; Department of Social Medicine, School of Medicine, University of Crete, Greece; Department of Social Medicine, School of Medicine, University of Crete, Greece; Department of Social Medicine, School of Medicine, University of Crete, Greece; Department of Psychology, School of Social Sciences and Humanities, University of Limassol, Cyprus; Department of Family Medicine, Faculty of Medicine, University of Ljubljana, Ljubljana, Slovenia; Department of Family Medicine, Faculty of Medicine, University of Ljubljana, Ljubljana, Slovenia; Faculty of Pharmacy, Institute of Clinical Pharmacy, University of Szeged; Albert Szent-Györgyi Health Centre, Department of Clinical Pharmacy, University of Szeged; Szeged Autumn Nursing Home, Szeged, Hungary; Centre for Family and Community Medicine, the Faculty of Health Sciences, Medical University of Lodz, Lodz, Poland; Department of Family Medicine, Lithuanian University of Health Sciences, Lithuania; Department of Clinical Pharmacology, Faculty of Medicine; Institute of Health Care Ethics, Faculty of Nursing and Health Professional Studies, Slovak Medical University, Bratislava, Slovakia; Department of Clinical Pharmacology, Faculty of Medicine; Institute of Health Care Ethics, Faculty of Nursing and Health Professional Studies, Slovak Medical University, Bratislava, Slovakia; Research Unit for Antibiotic Stewardship and Implementation, Department of Clinical Microbiology, Copenhagen University Hospital—Herlev and Gentofte, Herlev, Denmark

## Abstract

**Introduction:**

Nursing home residents frequently receive antibiotics, many of which may be unnecessary. This study aimed to develop an intervention applicable across European nursing homes to reduce antibiotic use by strengthening infection prevention and antimicrobial stewardship.

**Methods:**

The study ran January 2023 to December 2025. Using a participatory action research (PAR) approach, staff from participating nursing homes were engaged as project ambassadors and co-researchers, actively contributing to the development of the intervention. The study drew on quantitative and qualitative data and involved nursing homes in Denmark, Greece, Hungary, Lithuania, Poland, Slovakia, Slovenia and Spain.

**Results:**

A total of 109 nursing homes participated. We identified seven key topics: hand hygiene, personal protective equipment, indwelling urinary catheters, suspicion of urinary tract infection (UTI), high staff turnover and a lack of educated staff, relatives’ expectations and demands and insufficient knowledge of antibiotics. To cover these key topics, we developed 10 tools: 4 posters (hand hygiene, glove use, UTIs and basic information on viruses and bacteria); 3 leaflets (preventing UTIs, antibiotics and antimicrobial resistance and one for relatives); a hygiene quiz; a pocket card featuring a diagnostic UTI algorithm and a training programme for new staff. Educational lectures covering the seven key topics were also developed. Both the tools and the lectures were provided to the ambassadors during workshops in each country. The proportion of participating nursing homes adopting each tool ranged from 52% to 84%.

**Conclusions:**

The seven identified key topics are relevant to address to improve infection prevention and antimicrobial stewardship in European nursing homes.

## Introduction

Among nursing home residents, antibiotic use is frequent,^1,2^ making nursing homes reservoirs of resistant bacteria.^3–5^ Although this substantial use of antibiotics partly reflects residents’ frailty and vulnerability to infections^1,2^ antibiotic consumption remains significantly higher than in age- and comorbidity-matched community-dwelling populations.^6–8^ Suspected urinary tract infection (UTI) is the most common indication for antibiotic treatment,^9^ yet more than half of the treatments may be unnecessary because diagnostic criteria for UTI are not met.^10^ Reducing antibiotic use relies on both infection prevention and antimicrobial stewardship (AMS), the latter promoting appropriate antibiotic use in clinical practice.^1,11^

While infection prevention and control (IPC) guidelines are widely available and implemented in hospitals, they are often absent or inconsistently applied in nursing homes,^9,12,13^ and many nursing homes lack trained IPC staff and effective surveillance systems.^9^ Limited resources, high staff turnover and the challenge of maintaining a homelike care environment further complicate effective IPC implementation.^14–16^

AMS has also received greater attention in hospitals than in nursing homes, although its importance in the latter is increasingly recognized.^1,17^ The Centers for Disease Control and Prevention (CDC) developed the Core Elements of Antibiotic Stewardship for Nursing Homes, to guide stewardship activities in these settings.^1^ Compared with hospitals, AMS is more challenging to implement in nursing homes because prescribing physicians are only intermittently on site. While studies report mixed effects of AMS programmes in nursing homes, they appear particularly effective in reducing inappropriate antibiotic use for UTIs.^18–20^

Successful implementation of IPC and AMS interventions in nursing homes requires addressing the specific challenges of the setting, including workplace culture, staff attitudes and knowledge.^21^ The Participatory Action Research (PAR) approach provides a structured method for this. In PAR, intervention recipients are active partners in the research process, participating in a cyclical process of planning, action and reflection to collaboratively identify problems, implement solutions and evaluate outcomes, thereby supporting continuous improvement.^22,23^

This study aims to develop a multifaceted, PAR-informed intervention, suitable for diverse nursing home settings across Europe and designed to improve IPC and AMS processes, ultimately reducing unnecessary antibiotic use. To our knowledge, this is the first study to address an intervention targeting IPC and AMS simultaneously in the nursing home setting. Addressing both may be crucial for achieving effective reductions in antibiotic use.

## Methods

### Study context

This study was part of the ‘Improving Antibiotic Use in Long-Term Care Facilities by Infection Prevention and Control and Antibiotic Stewardship’ (IMAGINE) project.^24^ IMAGINE was a European Union (EU)-funded initiative running from January 2023 to December 2025, involving nursing homes from eight target countries: Denmark, Greece, Hungary, Lithuania, Poland, Slovakia, Slovenia and Spain.

The IMAGINE group consisted of researchers from multiple countries, including the eight countries with nursing homes participating in the project. The researchers had diverse professional backgrounds and expertise, including general practitioners, nursing home physicians, geriatricians, clinical microbiologists, clinical pharmacists, clinical pharmacologists, internists, public health specialists and infection control nurses.

### Study participants

From each participating country, we aimed to recruit 10–15 nursing homes through convenience sampling. The intervention primarily targeted nursing home staff, as they are directly involved in residents’ daily care, their IPC practices are critical and they strongly influence antibiotic use.^19^ Each participating nursing home selected approximately four staff members to serve as project ambassadors, acting as liaisons between the nursing homes and the research group and contributing to the development and implementation of the intervention. Staff members included nurses, nursing assistants and other personnel involved in direct resident care.

### Study design

The intervention was developed using a PAR approach, involving the ambassadors as co-researchers. The study is reported according to the Standards for Quality Improvement Reporting Excellence 2.0 guidelines^25^ to ensure clarity and completeness in reporting the design and the implementation (S1).

### The PAR process

**Figure 1.**
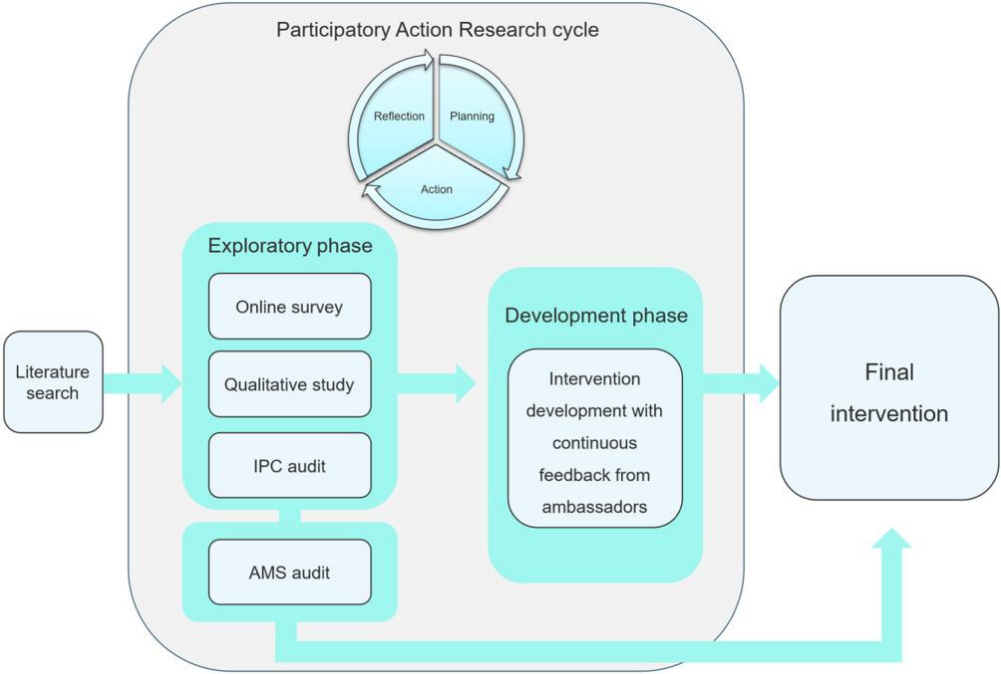
The PAR process.

Based on a literature search, IMAGINE partners initiated the exploratory phase aiming to pinpoint relevant focus areas and opportunities for improvement (Figure 1). A quantitative study (online survey) was developed and administered to nursing home staff to assess the conditions and availability of resources in the participating nursing homes. Next, a qualitative study using semi-structured interviews with nursing home staff members was conducted, focusing on their role in and perceived facilitators and barriers of IPC and AMS in the nursing home (S2).^16^ In addition, an audit chart with indicators relevant to IPC in nursing homes was developed. Each ambassador audited their nursing home by observing five colleagues performing care tasks and recorded their observations on the chart. Finally, an AMS audit (S3) was conducted, which recorded all antibiotic treatments in the included nursing homes between February and April 2024. The results of this audit were not available during the development of the intervention but were incorporated during the conduction of the intervention.^26^

After the exploratory phase, IMAGINE partners in each country met with national ambassadors to discuss the findings, either in person or online. Ambassadors provided feedback to inform intervention development, and country teams summarized the discussions. The development phase was iterative, with ambassadors regularly reviewing materials and suggesting improvements. Concurrently, IMAGINE partners synthesized input from all countries to develop a cohesive intervention. The intervention was initially developed in English and subsequently translated into the languages of the eight participating countries by the IMAGINE partners. Based on input from several countries, it was designed to be generic and applicable across settings, with minor contextual adaptations permitted.

Following the intervention, the ambassadors provided feedback on its utility.

### Ethics

The study adhered to the IMAGINE project protocol, the Declaration of Helsinki, Good Clinical Practice principles, the EU General Data Protection Regulation (2016/679), the Human Research Act, as well as other relevant local regulations. Appropriate regulatory/ethical approval was sought in each of the countries taking part in the study, and all study procedures started after gaining approval on the basis of the master protocol, translated where necessary to local language. The study protocol received ethical approval in Spain, the coordinating country, from the Ethics Committee of University Institute for Primary Health Care Research Jordi Gol i Gurina Foundation (IDIAPJGol) (ref. 23/080-P) on 12 July 2023.

## Results

In total, 109 nursing homes were included in the study, ranging from nine in Greece to 18 in Hungary. These nursing homes took part in the exploratory phase and the development phase that formed the foundation of the intervention.

### Exploratory phase

**Figure 2.**
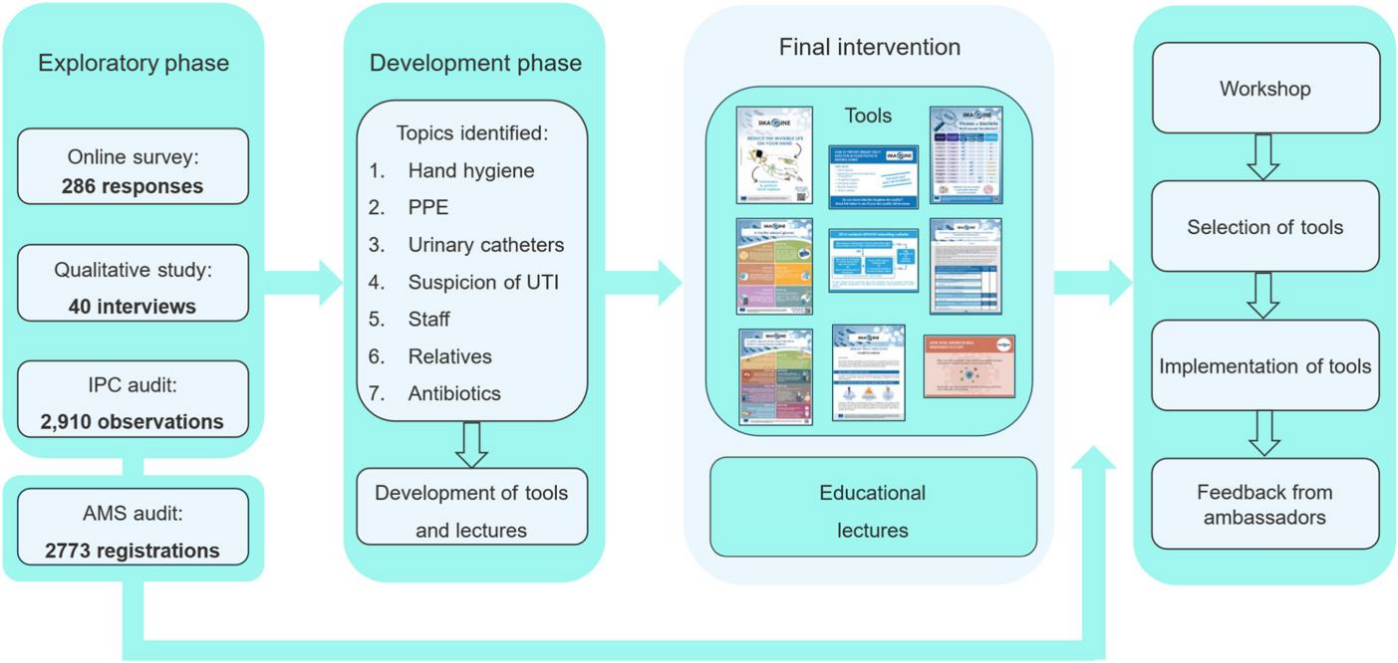
Overview of the results.

The online survey was completed by 286 nursing home staff members (Figure 2). The results revealed considerable variation both within but especially across countries regarding availability of equipment and resources, staff composition and education, as well as work procedures.

The qualitative study included 40 individual interviews with nursing home staff, 5 from each participating country. The findings indicated that staff often faced a challenging position, balancing respect for residents’ autonomy with the need to follow infection prevention guidelines. Regarding AMS, staff generally showed limited awareness of their important role in keeping a prudent antibiotic policy. Details of the qualitative study can be found elsewhere.^16^

The development of the IPC audit resulted in a chart containing 29 items on resource availability and procedure performance related to anogenital hygiene and the management of indwelling urinary catheters. The audit was conducted in all nursing homes, yielding 2910 observations of staff performing care tasks.

The AMS audit comprised 45 items addressing indication for treatment, risk factors for UTI, signs and symptoms, diagnostic testing and the choice and duration of antibiotic therapy. A total of 2773 antibiotic treatment episodes were recorded.

### Development phase

Based on the results of the exploratory phase, the IMAGINE partners and ambassadors jointly identified seven key topics to serve as the foundation for the development of the intervention. These topics were as follows:

1. Hand hygiene: when and how to perform it, and the impact of nail polish, artificial nails and jewellery.
2. Personal protective equipment (PPE): when and how to use gloves and aprons.
3. Indwelling urinary catheters: proper hygienic handling.
4. UTI suspicion: what should prompt suspicion and how to manage it.
5. Staff: high turnover and insufficiently trained staff.
6. Relatives: how staff can manage relatives’ expectations and demands regarding infection management and antibiotic prescribing.
7. Antibiotics: limited knowledge about their consequences and appropriate use.

Based on the identified key topics, several ideas for intervention materials were discussed, including videos and digital tools. However, there was consensus that printable materials would be most suitable for nursing home settings across the eight countries and most likely adaptable to other European contexts.

Besides printable materials, the ambassadors highlighted the need for staff education and training as an essential part of the intervention. Therefore, relevant educational lectures with PowerPoint slides were prepared by the IMAGINE partners, again based on the seven identified key topics.

### Final tools

In total, 10 printable materials—referred to as ‘tools’—were developed:

1. A poster about hand hygiene.
2. A hygiene quiz, accessible through a QR code located on the poster above (Tool 1).
3. A poster about glove use.
4. A leaflet with information on how to prevent UTIs.
5. A poster addressing common myths about UTIs (signs and symptoms, dipstick testing, prevention, etc.).
6. A leaflet for relatives with information about UTIs.
7. A pocket card with a diagnostic UTI algorithm.
8. A training programme for new staff with information on how to prevent UTIs.
9. A poster with basic information on viruses and bacteria.
10. A leaflet about antibiotics and antimicrobial resistance.

The 10 tools, available in all 8 IMAGINE languages plus English, can be downloaded from the IMAGINE homepage.^27^

### Rollout of the intervention

The intervention was delivered through workshops conducted in all countries. Ambassadors, nursing home managers and other interested staff from participating nursing homes were invited. Workshops were planned as 2 day, in-person events; however, some countries held 1 day workshops or conducted parts online to facilitate participation. The overall programme was consistent across countries but allowed adaptation to national and local conditions. The number of participating nursing home staff members ranged from 14 to 65 across countries, with a mean of 35 participants per country.

During the workshops, the educational lectures were delivered by IMAGINE partners or local experts. The lectures incorporated AMS audit findings, including analyses of treatment appropriateness and documented signs and symptoms of UTI.^26^ Participants were introduced to the final tools and selected those they wished to implement in their nursing homes. As the tools were pre-printed, they could be used immediately. Implementation strategies were discussed, and participants were encouraged to share their knowledge with colleagues. Lecture slides were subsequently distributed by email.

After the workshops, the IMAGINE partners were available by phone and email to the ambassadors, providing additional prints of the tools and feedback as needed. In addition, ambassadors could request tools other than those they had initially selected during the workshop.

**Figure 3.**
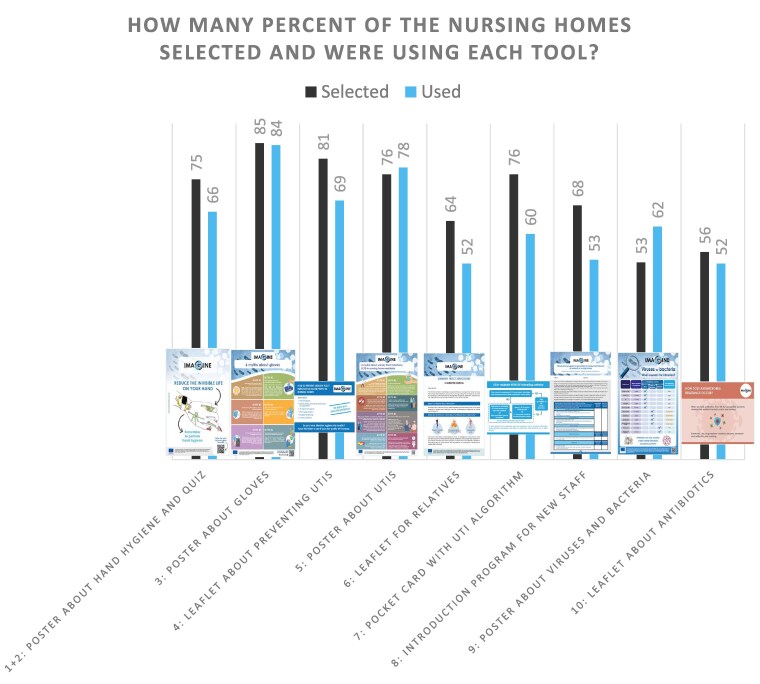
Percentage of participating nursing homes that selected and used each tool. Note: The nursing homes were able to adopt additional tools beyond those selected during the workshop. This explains why a higher proportion reported using Tool 5 and Tool 9 compared with the proportion that initially selected it.

One to three months after the workshop, a questionnaire (S4) was sent to each nursing home asking the ambassadors which tools they were still using (Figure 3). Tool 3 (poster about gloves) was reported as being used most frequently (by 84% of the nursing homes), whereas Tool 6 (leaflet for relatives) and Tool 10 (leaflet about antibiotics) were reported as being used least often (by 52% of the nursing homes).

As shown in Table 1, the countries were generally aligned regarding which tools were most frequently used, although some variation was observed.

**Table 1.** Proportion of nursing homes in each country using each tool, assesed 1–3 months after the workshop

	Tool number								
	1 + 2 Poster about hand hygiene and quiz (%)	3 Poster about gloves (%)	4 Leaflet about preventing UTIs (%)	5 Poster about UTIs (%)	6 Leaflet for relatives (%)	7 Pocket card with UTI algorithm (%)	8 Introduction programme for new staff (%)	9 Poster about viruses and bacteria (%)	10 Leaflet about antibiotics (%)
Denmark	33	47	40	67	27	47	33	20	13
Greece	44	67	78	67	56	56	89	67	78
Hungary	89	89	67	83	67	72	44	83	61
Lithuania	73	100	80	87	87	60	47	73	80
Poland	64	100	82	82	36	73	64	73	73
Slovakia	60	100	60	60	50	40	100	80	50
Slovenia	90	100	60	100	20	70	40	70	50
Spain	69	77	92	77	62	69	38	38	23
Total	66	84	69	78	52	60	53	62	52

When asked to identify the three most useful tools, Tools 3, 5 and 4 were selected—in that order—which, as seen in Table 1, were also the three most frequently used tools. This question was designed to encourage staff to rank the tools and provide feedback on which they found most valuable, even if they had selected all of them.

Finally, the nursing homes were asked about the implementation process at their facilities. Overall, the responses suggested that the nursing homes had not yet had sufficient time to fully implement the tools, but that the implementation process was well underway (S4).

## Discussion

### Main findings

This study describes the development of a multifaceted intervention applicable across diverse European nursing home settings. We identified seven key topics that are relevant to focus on to improve IPC and AMS in nursing homes. Based on these findings, 10 tools were developed that are broadly applicable to nursing home staff, as a high proportion of nursing homes across the eight participating countries selected and used each tool. All 10 tools were ranked among the 3 most useful by at least some nursing homes, indicating that all the tools were considered relevant and useful. The intervention phase was generally reported by the nursing home staff to be positive, though challenged by workload pressures and limited resources.

### Interpretation and comparison with existing literature

The PAR approach in this study builds on previous applications in nursing homes. van Buul *et al.*^28^ used PAR in the IMPACT study to tailor AMS interventions in Dutch nursing homes based on local data and stakeholder input. Hartman *et al.*^29^ similarly applied a modified PAR approach in the ImpresU study to implement a multifaceted AMS intervention for UTIs across European settings. The present study extends the PAR approach by integrating both IPC and AMS components across multiple European nursing homes. In addition, this study places stronger emphasis on co-development of implementation tools and adaptation across diverse organizational and national contexts. This broadens the scope of earlier PAR work, which primarily focused on AMS within more limited geographical or clinical settings.

Hand hygiene was an essential topic in the exploratory phase and was addressed in Tools 1, 2, 4 and 8. A systematic review from 2015^30^ assessed the impact of hand hygiene interventions on infection risk and found that 35 of 56 included studies (63%) reported reductions in infections among residents and/or staff. A randomized controlled trial from 2021^31^ showed that a multimodal hand hygiene intervention improved compliance among nursing home staff; however, it did not reduce infections among residents, and no statistically significant difference in UTI incidence was observed.^32^

PPE, including gloves and aprons, was identified as a relevant topic in our study and addressed in Tools 3, 4, 5 and 8. PPE use in nursing homes has been controversial due to concerns about stigmatization and increased psychological distance from residents,^33,34^ a dilemma we also observed during our exploratory phase. Evidence on the effect of PPE is limited but suggests potential benefits: a pilot study from 2021 reported reduced resident-to-resident transmission of Staphylococcus *aureus* after a 2 month intervention in which staff wore aprons and gloves during high-risk care activities.^34^ The CDC also provides recommendations for PPE use in nursing homes.^35^ The high uptake of PPE-related tools in our study reflects its relevance in daily practice. Despite concerns about relational consequences, the widespread use of these tools highlights the need for practical, context-sensitive approaches. Overall, PPE use in nursing homes remains unresolved and warrants further research to balance infection prevention with person-centred care.

Topic 3, indwelling urinary catheters, was addressed by Tools 4, 5, 7 and 8. A study from 2017 including 404 nursing homes reported a 54% reduction in catheter-associated UTIs (CAUTIs) following a multifaceted intervention targeting catheter removal, aseptic insertion, regular reassessment of catheter need, staff training and incontinence care planning.^36^ The project also developed an online toolkit with posters, slides and videos to reduce CAUTIs.^37^ A systematic review published in 2017 identified interventions such as reducing catheter use, improving catheter care, enhancing hand hygiene and strengthening IPC measures as potentially effective, though the evidence was limited by small samples and methodological heterogeneity.^38^

Nursing home staff reported difficulty determining whether residents had a UTI, making the management of suspected UTI a key topic. This was addressed in Tool 7, a pocket card for quick reference that included an algorithm to assess the likelihood of UTI, based on established decision tools such as the McGeer and Loeb criteria, which have been shown to reduce antibiotic use in nursing homes.^10,39,40^ The algorithm was designed as a simplified, user-friendly version of existing evidence-based tools. Adapting such algorithms into practical formats may help bridge the gap between guidelines and clinical decision-making and support AMS efforts in nursing homes.

Topic 5, staff, was primarily addressed by Tool 8. Staff turnover in nursing homes is high, often exceeding that in other healthcare settings, and many staff lack formal healthcare education.^41,42^ Participating staff noted that some colleagues lacked essential IPC and AMS knowledge, which was the focus of Tool 8. The CDC’s Core Elements of Antibiotic Stewardship for Nursing Homes^1^ emphasize structured education for all staff, including interactive workshops and ongoing training, though not specifically for newly employed or untrained personnel.

Relatives were identified as an important topic, as staff reported that they sometimes pressure for diagnostic testing and antibiotics, complicating stewardship—an issue highlighted in the qualitative study.^16^ A systematic review from 2022 found that relatives’ expectations often persist even after staff-focused educational interventions.^43^ To address this, we developed a leaflet for relatives (Tool 6) with key information on UTIs and antibiotic use, with additional related content included in Tools 1, 2, 3, 5, 9 and 10.

The final topic identified was staff knowledge of antibiotics. AMS was less prominent than IPC, likely because the staff members saw prescribing as the physician’s responsibility, while themselves feeling more accountable for IPC. Accordingly, tools addressing antibiotic knowledge were limited to Tools 9 and 10. Nevertheless, their inclusion is important, as the CDC recommends AMS education for all nursing home staff, and evidence shows it can substantially improve prescribing practices.^1^

In addition to the tools, educational lectures were a key part of the intervention, covering all seven key topics. Systematic reviews show that multifaceted stewardship programmes combining education and tools are most consistently associated with reduced antibiotic use.^44–46^

### Strengths and limitations

A key strength of this study is the PAR approach, which actively involved nursing home staff from eight European countries in developing the intervention. This co-creation enhanced contextual relevance and acceptability, ensuring the intervention was grounded in the everyday practices, constraints and priorities of staff. Including a large number of nursing homes across diverse healthcare systems further strengthens its applicability across European nursing home settings.

Several limitations should be acknowledged. First, the participatory, multinational development process required balancing country-specific priorities with the goal of creating an internationally applicable intervention. Consequently, some topics highly relevant in individual countries may have been excluded if not shared across settings, while others included may have had limited relevance locally. This reflects an inherent trade-off between local specificity and cross-national applicability.

In addition, the use of PAR may have introduced selection bias, as staff who agreed to serve as ambassadors were likely more motivated or engaged in IPC and AMS than non-participants, meaning the findings may not fully reflect the perspectives of all nursing home staff. Resource and time constraints also limited staff involvement and hindered an optimal PAR process; more continuous engagement, including additional meetings with ambassadors, would have been desirable. Several ambassadors reported difficulties finding time to introduce the intervention in their nursing homes and to engage colleagues with the tools.

Finally, in the project, the staff chose which intervention materials to implement in their nursing home. A potential drawback of this approach is that it may favour the selection of the easiest, least invasive and therefore possibly least effective interventions, as proposed by van Buul *et al*.^28^ This may have occurred in our study, where the results show that the posters are used relatively more than the other tools (Figure 3). The impact of a poster alone is questionable, particularly if it is not accompanied by education explaining the underlying rationale.

### Implications and future research

The intervention provides a flexible, staff-focused resource to support IPC and AMS in nursing homes. Its modular structure allows nursing homes across diverse settings to select and adapt tools according to local priorities and resources. Several components may be particularly useful for staff education and the training of new staff, including in settings with high staff turnover. This study describes the development process, whereas the intervention’s impact was not evaluated. Future research should assess its effects on IPC outcomes and antibiotic prescribing, as well as examine sustainability and long-term use.

As the intervention was conducted as a bundled approach, with multiple materials introduced concurrently, it is not possible to evaluate the contribution of individual components. Future studies may therefore benefit from exploring the effectiveness of specific intervention materials or subsets of tools, either alone or in combination, to better understand which components are most influential in different nursing home contexts.

### Conclusions

Our results demonstrate that the seven IPC and AMS topics identified, and the corresponding tools developed through the PAR approach, were relevant to staff in European nursing homes, as all tools were implemented by the majority of the participating nursing homes.

## Supplementary Material

dlag158_Supplementary_Data

## Supplementary data

Supplementary S1–S4 are available as Supplementary data at *JAC-AMR* Online.
