# Supplementary material for: Development of a participatory action research–based intervention to enhance infection prevention and antimicrobial stewardship among European nursing home staff
Source: JAC Antimicrob Resist. 2026 Aug 12;8(4):dlag158. doi: 10.1093/jacamr/dlag158 (PMC13463765; doi:10.1093/jacamr/dlag158)
Supplement: Supplementary file 1 [file dlag158_supplementary_data.docx]

SUPPLEMENTARY 1 (S1)

| SQUIRE 2.0 Checklist | | | |
| --- | --- | --- | --- |
| No. | **SQUIRE item** | **Description of requirement** | **Reported in manuscript** |
| 1 | Title | Indicate that the manuscript concerns an initiative to improve healthcare (broadly defined to include the quality, safety, effectiveness, patient centeredness, timeliness, cost, efficiency, and equity of healthcare) | Line 1 |
| 2 | Abstract | a. Provide adequate information to aid in searching and indexing  b. Summarize all key information from various sections of the text using the abstract format of the intended publication or a structured summary such as: background, local problem, methods, interventions, results, conclusions | Lines 65-92 |
| 3 | Problem description | Nature and significance of the local problem | Lines 115-122 |
| 4 | Available knowledge | Summary of what is currently known about the problem, including relevant previous studies | Lines 123-134 |
| 5 | Rationale | Informal or formal frameworks, models, concepts, and/or theories used to explain the problem, any reasons or assumptions that were used to develop the intervention(s), and reasons why the intervention(s) was expected to work | Lines 135-141 |
| 6 | Specific aims | Purpose of the project and of this report | Lines 142-146 |
| 7 | Context | Contextual elements considered important at the outset of introducing the intervention(s) | Not relevant |
| 8 | Intervention(s) | a. Description of the intervention(s) in sufficient detail that others could reproduce it  b. Specifics of the team involved in the work | a: Lines 178-199  b: Lines 155-159 |
| 9 | Study of the intervention(s) | a. Approach chosen for assessing the impact of the intervention(s)  b. Approach used to establish whether the observed outcomes were due to the intervention(s) | Not applicable, as the study addresses the development of an intervention rather than its impact |
| 10 | Measures | a. Measures chosen for studying processes and outcomes of the intervention(s), including rationale for choosing them, their operational definitions, and their validity and reliability  b. Description of the approach to the ongoing assessment of contextual elements that contributed to the success, failure, efficiency, and cost  c. Methods employed for assessing completeness and accuracy of data | Not applicable, as the study addresses the development of an intervention rather than its impact |
| 11 | Analysis | a. Qualitative and quantitative methods used to draw inferences from the data  b. Methods for understanding variation within the data, including the effects of time as a variable | Not applicable, as the study addresses the development of an intervention rather than its impact |
| 12 | Ethical considerations | Ethical aspects of implementing and studying the intervention(s) and how they were addressed, including, but not limited to, formal ethics review and potential conflict(s) of interest | Lines 204 – 211. |
| 13 | Results | a. Initial steps of the intervention(s) and their evolution over time (e.g., time-line diagram, flow chart, or table), including modifications made to the intervention during the project  b. Details of the process measures and outcome  c. Contextual elements that interacted with the intervention(s)  d. Observed associations between outcomes, interventions, and relevant contextual elements  e. Unintended consequences such as unexpected benefits, problems, failures, or costs associated with the intervention(s).  f. Details about missing data | Lines 214 – 316. Not all items are applicable. |
| 14 | Summary | a. Key findings, including relevance to the rationale and specific aims  b. Particular strengths of the project | a. Lines 320-328.  b. Lines 401-406. |
| 15 | Interpretation | a. Nature of the association between the intervention(s) and the outcomes  b. Comparison of results with findings from other publications  c. Impact of the project on people and systems  d. Reasons for any differences between observed and anticipated outcomes, including the influence of context  e. Costs and strategic trade-offs, including opportunity costs | Lines 331-398. |
| 16 | Limitations | a. Limits to the generalizability of the work  b. Factors that might have limited internal validity such as confounding, bias, or imprecision in the design, methods, measurement, or analysis  c. Efforts made to minimize and adjust for limitations | Lines 407-425. |
| 17 | Conclusions | a. Usefulness of the work  b. Sustainability  c. Potential for spread to other contexts  d. Implications for practice and for further study in the field e. Suggested next steps | Lines 428-445. |
| 18 | Funding | Sources of funding that supported this work. Role, if any, of the funding organization in the design, implementation, interpretation, and reporting | Lines 452-457 |

SUPPLEMENTARY 2 (S2)

SUPPLEMENTARY 3 (S3)

SUPPLEMENTARY 4 (S4)

Dear IMAGINE participant.

We would like to know how the implementation process went in your nursing home. Please help us by answering the four questions below.

| 1. Which tools are you still using in your nursing home? (Please tick off) | | | | | | | | |
| --- | --- | --- | --- | --- | --- | --- | --- | --- |
| 1 + 2 | 3 | 4 | 5 | 6 | 7 | 8 | 9 | 10 |
| Poster and quiz | Poster about gloves | Leaflet about UTI | Poster about UTI | Leaflet for relatives | UTI pocket card | Training program for new staff | Poster about antibiotics | Leaflet about antibiotics |
|  |  |  |  |  |  |  |  |  |
|  |  |  |  |  |  |  |  |  |

2. In your opinion, which three tools have been most useful in your nursing home?

(Please write the number of the tool)

|  | Most useful tool: |  | Second most useful tool: |  | Third most useful tool: |
| --- | --- | --- | --- | --- | --- |

3. Overall, how did the implementation process go in your nursing home? (Please describe)

________________________________________________________________________________________________________________________________________________________________________________________________________________________________________________________________________________________________________________________________________________________________________________________________________________________________________________________________________________________________________________________________________

4. For the tools you are still using, how did you implement them? (Please describe in the form below)

| Tool number |  | Implementation |
| --- | --- | --- |
| 1+2 | Poster and quiz |  |
| 3 | Poster about gloves |  |
| 4 | Leaflet about UTI |  |
| 5 | Poster about UTI |  |
| 6 | Leaflet for relatives |  |
| 7 | UTI pocket card |  |
| 8 | Training program for new staff |  |
| 9 | Poster about antibiotics |  |
| 10 | Leaflet about antibiotics |  |

Other comments? _____________________________________________________________________________________________________________________________________________________________________________________________________________________________________________________________________
